# Knockdown of Two Trehalase Genes by RNA Interference Is Lethal to the White-Backed Planthopper *Sogatella furcifera* (Horváth) (Hemiptera: Delphacidae)

**DOI:** 10.3390/biom12111699

**Published:** 2022-11-17

**Authors:** Zhao Wang, Gui-Yun Long, Dao-Chao Jin, Hong Yang, Cao Zhou, Xi-Bin Yang

**Affiliations:** 1 College of Environment and Life Sciences, Kaili University, Kaili 556011, China; 2 Provincial Key Laboratory for Agricultural Pest Management of Mountainous Regions, and Scientific Observing and Experimental Station of Crop Pests in Guiyang, Ministry of Agriculture and Rural Affairs of the People’s Republic of China, Institute of Entomology, Guizhou University, Guiyang 550025, China; 3 School of Ethnic Medicine, Guizhou Minzu University, Guiyang 550025, China; 4 College of Life Sciences, Chongqing Normal University, Chongqing 401331, China

**Keywords:** *Sogatella furcifera*, trehalase, gene expression, RNA interference

## Abstract

Trehalase (Tre) is a crucial enzyme involved in trehalose metabolism, and it plays pivotal roles in insect development and metamorphosis. However, the biological function of *Tre* genes in *Sogatella furcifera* remains unclear. In the present study, two *Tre* genes—*SfTre1* and *SfTre2*—were cloned and identified based on the *S. furcifera* transcriptome data. Bioinformatic analysis revealed that the full-length complementary DNA of *SfTre1* and *SfTre2* genes were 3700 and 2757 bp long, with 1728- and 1902-bp open reading frame encoding 575 and 633 amino acid residues, respectively. Expression analysis indicated that *SfTre1* and *SfTre2* were expressed at all developmental stages, with the highest expression in day two adults. Furthermore, the highest expression levels of *SfTre1* and *SfTre2* were observed in the ovary; enriched expression was also noted in head tissues. The knockdown of *SfTre1* and *SfTre2* via injecting double-stranded RNAs decreased the transcription levels of the corresponding mRNAs and led to various malformed phenotypes and high lethality rates. The results of our present study indicate that *SfTre1* and *SfTre2* play crucial roles in *S. furcifera* growth and development, which can provide referable information for *Tre* genes as a potential target for planthopper control.

## 1. Introduction

Rice (*Oryza sativa* L.) is an important food crop worldwide. More than 50% of the world’s population relies on rice as their staple food. Particularly in Asia, rice and rice farming are important to lifestyle, cultural heritage, customs, and spiritual beliefs [1]. However, rice production has long been threatened by various insect pests. The white-backed planthopper *Sogatella furcifera* (Horváth) (Hemiptera: Delphacidae), a notorious migratory insect, is a destructive rice insect pest in some Asia-Pacific countries [2,3]. This pest directly damages rice by sucking phloem sap and ovipositing [4,5,6], as well as transmitting viruses, such as the southern rice black-streaked dwarf virus [7,8]. To date, the use of chemical pesticides has been the primary strategy for *S. furcifera* management [9]. However, the continued use of insecticides has led to the development of insect resistance and environmental issues [10,11]. Therefore, there is an urgent need to develop efficient and environmentally friendly methods for controlling *S. furcifera* population.

In insects, molting involves the shedding of the old epidermis and the production of the new epidermis, as well as the degradation and resynthesis of chitin. Insect chitin biosynthesis is a complex physiological and biochemical process. Studies have reported that the insect chitin biosynthesis pathway begins with the hydrolysis of trehalose to glucose by trehalases [12]. It is well established that the trehalose biosynthesis pathway in insects involves trehalose-6-phosphate synthase (TPS) and trehalose-6-phosphate phosphatase (TPP) [13]. TPS catalyzes the transfer of glucose from uridine diphosphate -glucose to glucose-6-phosphate for forming trehalose-6-phosphate, whereas TPP dephosphorylates trehalose-6-phosphate to trehalose predominantly in the fat body, which is then released into the hemolymph. Following the absorption of trehalose by trehalose-utilizing cells, it is hydrolyzed into glucose by trehalase (Tre); this is the only known pathway of trehalose utilization [14].

Tre (EC 3.2.1.28) is a glycosidase that is commonly found in bacteria, fungi, plants, and animals [15,16]. It catalyzes trehalose to produce two molecules of glucose, which are then used for various physiological and life activities [17,18,19]. Tre was first isolated from *Aspergillus niger* in 1893 by Bourquelot; its corresponding role was then reported in *Saccharomyces cerevisiae* [14]. In insects, two forms of Tre were found by protein purification and enzyme activity determination: soluble trehalase (Tre1) and membrane-bound trehalase (Tre2). The first soluble Tre complementary DNA (cDNA) sequence was cloned from *Tenebrio molitor* [20]. To date, at least 20 insect Tre cDNA sequences have been identified and cloned, including *Pimpla hypochondriaca* [21], *Apis mellifera* [22], *Bombyx mori* [23], *Nilaparvata lugens* [24], *Harmonia axyridis* [25], *Apolygus lucorum* [26], *Chironomus ramosus* [27], and *Anopheles stephensi* [28]. Studies have found that although the amino acid sequence similarity of the two types of Tre in insects is extremely low, they have some common characteristics in protein structure. For example, the two tag sequences PGGRFx(R/I)Ex(L/F)YYWDx(T/S)Y and QWDx(F/Y)PNx(S/A/V)Wx(A/P)P, and a glycine-rich region GGGGEY. In addition, compared with Tre1, Tre2 in the C-terminus often includes a hydrophobic transmembrane domain of approximately 20 amino acids [23,29]. A recent study found that *Laodelphax striatellus* Tre2 contains two varying transmembrane domains [30]. These phenomena suggest that they potentially serve different functions in organisms.

Based on previous reports, Tre provides energy for the growth and development of insects and regulates the transcription of chitin biosynthesis-related genes, thereby influencing chitin formation. In *H. axyridis*, the genes *TRE2-like* and *TRE2* were silenced, which led to a significant decrease in the expression levels of chitin synthase genes (*CHSA* and *CHSB*), thereby resulting in developmental defects and mortality in adults [31]. Similarly, in *Glyphodes pyloalis*, the knockdown of *GpTre1* and *GpTre2* significantly hindered the expression levels of *GpCHSA* and *GpCHSB*, which caused issues during larval molting and resulted in abnormal adult wing development [32]. A previous study reported that the formation of new cuticular chitin in *Locusta migratoria* demands vast quantities of Tre, and the knockdown of this gene disturbs the chitin biosynthesis of the insect epidermis [33]. Being the first essential enzyme in the insect chitin biosynthesis pathway, Tre can be used as a potential target for controlling insect pests.

In our previous research, chitin biosynthesis genes, including UDP-*N*-acetylglucosamine pyrophosphorylase (*SfUAP*) and chitin synthase (*SfCHS*), have been characterized in *S. furcifera* [34,35]. However, there is no information on the *trehalase* genes involved in the chitin synthesis pathway in *S. furcifera*. In the present study, we cloned and characterized two *Tre* genes—*SfTre1* and *SfTre2*—and investigated changes in their expression levels at different developmental stages and in different tissues of *S. furcifera*. In addition, we explored the function of *SfTre1* and *SfTre2* genes using RNA interference (RNAi) technology. The results of this study could provide molecular insights into the biological functions of *SfTre1* and *SfTre2* in the molting and development of *S. furcifera* and give information regarding their application potential as candidate genes for pest control.

## 2. Materials and Methods

### 2.1. Insect Rearing

The *S. furcifera* specimens used in the present study were collected in 2013 from rice fields in Huaxi District, Guiyang City, Guizhou Province, China (26°31′302″ N, 106°62′294″ E). They were maintained in an insectary using a previously described method [35].

### 2.2. Primer Design

The fragment sequences of *SfTre1* and *SfTre2* genes were screened from the transcriptome sequencing data of *S. furcifera* (SRR116252), and their primers were designed using Primer Premier 6.0 software (Palo Alto, CA, USA). Primers are shown in Table 1. They were synthesized by Sangon Biotech Co. Ltd. (Shanghai, China).

### 2.3. Total RNA Extraction and cDNA Synthesis

Total RNA was isolated from the whole body of *S. furcifera* nymphs or adults using the HP Total RNA Kit (Omega Bio-Tek, Norcross, GA, USA) with genomic DNA removal columns, according to the manufacturer’s instructions. Total RNA quality was detected using 1% agarose gel electrophoresis, and RNA concentration was determined using a Nanodrop 2000 spectrophotometer (Thermo Fisher Scientific, Wilmington, DE, USA). The purified RNA was stored at −80 °C until future use. First-strand cDNA was synthesized from 2 μg of total RNA using the AMV First Strand cDNA Synthesis Kit (Sangon Biotech Co. Ltd., Shanghai, China) with an oligo(dT) primer, according to manufacturer’s instructions and stored at −20 °C until use.

### 2.4. SfTre1 and SfTre2 Gene Cloning

The cDNA fragment-encoding sequences of *SfTre1* and *SfTre2* sequences were amplified by polymerase chain reaction (PCR) using *S. furcifera* cDNA. PCR amplifications were performed using the primer pairs listed in Table 1 and LA Taq^®^ polymerase (TaKaRa Biotechnology, Dalian, China). Briefly, 1 μL of each primer (10 μM), 2.5 μL of 10× LA PCR buffer (Mg^2+^ plus), 4 μL of dNTP mixture (2.5 mM), 1 μL of cDNA template, 0.25 μL of LA Taq polymerase, and 15.25 μL of double-distilled water were added to a tube to a final volume of 25 μL. Thermal cycling was conducted using a T100 Thermal Cycler (Bio-Rad, Hercules, CA, USA) according to the following conditions: initial denaturation at 94 °C for 3 min, followed by 30 cycles of denaturation at 94 °C for 30 s, annealing at 52–58 °C (according to primer annealing temperature) for 30 s, extension at 72 °C for 1–3 min (according to the amplified fragment size); and a final extension at 72 °C for 10 min. The PCR products were excised through 1% agarose gel electrophoresis and purified using the EasyPure^®^ Quick Gel Extraction Kit (TransGen Biotech, Beijing, China). Thereafter, the purified products were ligated into the pMD-18T vector (TaKaRa Biotechnology, Dalian, China) and submitted to the Sangon Corporation for assessing the validity of the sequences.

Full-length cDNAs of *SfTre1* and *SfTre2* were obtained using the rapid amplification of cDNA ends PCR (RACE-PCR). The SMARTer^®^ RACE 5′/3′ Kit (Clontech, Mountain View, CA, USA) was used to amplify the 5′ and 3′ ends of *SfTre1* and *SfTre2*, according to the manufacturer’s instructions. In particular, the amplification conditions for the primary RACE-PCR using the long universal primer and gene-specific primers (GSPs, Table 1) were as follows: 30 cycles of denaturation at 94 °C for 30 s, annealing at 54–56 °C (according to primer annealing temperature) for 30 s, and extension at 72 °C for 60 s. For the nested PCR reaction, the primary PCR product was initially diluted 100 times and then used as a template with the short universal primer and GSPs. The parameters of the nested PCR reaction were the same as those of the primary PCR reaction. The 5′-RACE products were purified and sequenced as previously described.

### 2.5. Sequence Analysis

We used SeqMan software to assemble sequenced fragments for obtaining the full-length cDNA of *SfTre1* and *SfTre2*. DNAMAN 7.0 (Lynnon Biosoft, California, CA, USA) was used to edit nucleotide sequences. A similarity search and homology comparison were performed using the NCBI BLAST program (https://blast.ncbi.nlm.nih.gov/Blast.cgi, accessed on 25 August 2019). The physical and chemical properties of the protein were analyzed using the NCBI Open Reading Frame (ORF) Finder (https://www.ncbi.nlm.nih.gov/orffinder/, accessed on 29 August 2019), ExPASy ProtParam tool (https://web.expasy.org/protparam/, accessed on 29 August 2019), NetNGlyc 1.0 (https://services.healthtech.dtu.dk/service.php?NetNGlyc-1.0, accessed on 29 August 2019), TMHMM v.2.0 (https://services.healthtech.dtu.dk/service.php?TMHMM-2.0, accessed on 29 August 2019), and SignalP 4.1 (http://www.cbs.dtu.dk/services/SignalP/, accessed on 29 August 2019). The three-dimensional (3D) models of *SfTre1* and *SfTre2* were predicted using the SWISS-MODEL program (https://www.swissmodel.expasy.org/interactive, accessed on 5 September 2019) and then visualized using the PyMOL Molecular Graphics System 1.1. A phylogenetic tree was constructed using MEGA 6.06 by the neighbor-joining method, and bootstrap analyses of 1000 replications were performed.

### 2.6. Quantitative Real-Time PCR (qRT-PCR)

As described previously [34,35], *S. furcifera* were sampled from different stages ranging from eggs to adults, to investigate the developmental stage expression levels. Five tissue samples that involved the integument, fat body, gut, head, and ovary were dissected from day one fifth-instar nymphs and day three adults to detect tissue-specific expression. These experiments were performed in triplicates. The total RNA of each sample was isolated using the HP Total RNA Kit (with genomic DNA removal columns; Omega Bio-Tek, Norcross, GA, USA), and first-strand cDNA synthesis was performed using the AMV RT Reagent Kit with an oligo(dT) primer (Sangon Biotech Co. Ltd., Shanghai, China). The GSPs used for qRT-PCR are shown in Table 1. The *S. furcifera* 18S rRNA was chosen as the control gene. The expression levels of *SfTre1* and *SfTre2* were estimated by qRT-PCR using the CFX-96 real-time qPCR system (Bio-Rad) and FastStart Essential DNA Green Master (Roche Diagnostics, Shanghai, China). Each reaction mixture contained 1 μL of each primer (10 μM), 1 μL of cDNA sample, 10 μL of FastStart Essential DNA Green Master, and 7 μL of RNase-free water to make a final volume of 20 μL. The qPCR cycling parameters were as follows: initial denaturation at 95 °C for 10 min, followed by 40 cycles of denaturation at 95 °C for 30 s, and annealing at 55 °C for 30 s. Finally, the melting curve analysis was performed from 65 °C to 95 °C. The relative gene expression levels were calculated using the 2^−ΔΔCt^ method [36].

### 2.7. SfTre1 and SfTre2 Gene Silencing and Phenotypes

Gene silencing was performed by injecting day one fifth-instar *S. furcifera* nymphs with double-stranded RNA (dsRNA) to explore the biological roles of *SfTre1* and *SfTre2*. The unique cDNA region of *SfTre1* and *SfTre2* was targeted for dsRNA synthesis, and green fluorescent protein (GFP) was used as the control. The primers used for the experiment contained the T7 RNA polymerase promoter (Table 1). MEGAscript^®^ RNAi Kit (Ambion, Carlsbad, CA, USA) was used to synthesize dsRNA. Then, the dsRNA products were purified using the EasyPure^®^ Quick Gel Extraction Kit (TransGen Biotech, Beijing, China). Subsequently, the size of the purified products was determined using 1% agarose gel electrophoresis, and the concentration of dsRNA was determined with a Nanodrop 2000 spectrophotometer (Thermo Fisher Scientific, Wilmington, DE, USA).

The Nanoliter 2010 microinjector (World Precision Instruments, Sarasota, FL, USA) was used as described previously [34,35]. The ventral side of the prothorax and mesothorax of *S. furcifera* was chosen as the injection point, and 100 ng of dsRNAs targeting the two *SfTre* sequences and GFP was injected into each nymph. Overall, 50 nymphs from each group were injected, and the experiment was performed in triplicate. Injected nymphs were reared on fresh rice seedlings, and their death was recorded every 12 h for five consecutive days. Meanwhile, nymphs with visible misshapen phenotypes were photographed under a Keyence VH-Z20R stereoscopic microscope (Keyence, Osaka, Japan). At 72 h after injection, eight nymphs were randomly selected from each group to detect the mRNA expression levels of *SfTre1* and *SfTre2* using qRT-PCR.

### 2.8. Statistical Analysis

The mRNA expression levels of *SfTre1* and *SfTre2* at different developmental stages and in various tissues were calculated using a one-way analysis of variance. A *p*-value of <0.05 or 0.01 was considered significant or highly significant, respectively, in Duncan’s multiple-range test. An Independent sample *t*-test was used to evaluate the significance of gene silencing. All analyses were conducted using SPSS 13.0 software (IBM Inc., Chicago, IL, USA).

## 3. Results

### 3.1. SfTre1 and SfTre2 Sequence Analysis

Based on the transcriptome database of *S. furcifera*, two *Tre* genes—*SfTre1* and *SfTre2*—were screened. Then, the full-length cDNA sequence of these two genes was cloned and verified using multiplex PCR and RACE. Sequence analysis indicated that the 1728-bp ORF of *SfTre1* encodes a protein of 575 amino acid residues with a molecular weight of 67.01 kDa and an isoelectric point of 5.44 (Table 2). Meanwhile, the 1902-bp ORF of *SfTre2* encodes a protein of 633 amino acid residues with a molecular weight of 72.82 kDa and an isoelectric point of 5.65 (Table 2).

**Figure 1:**
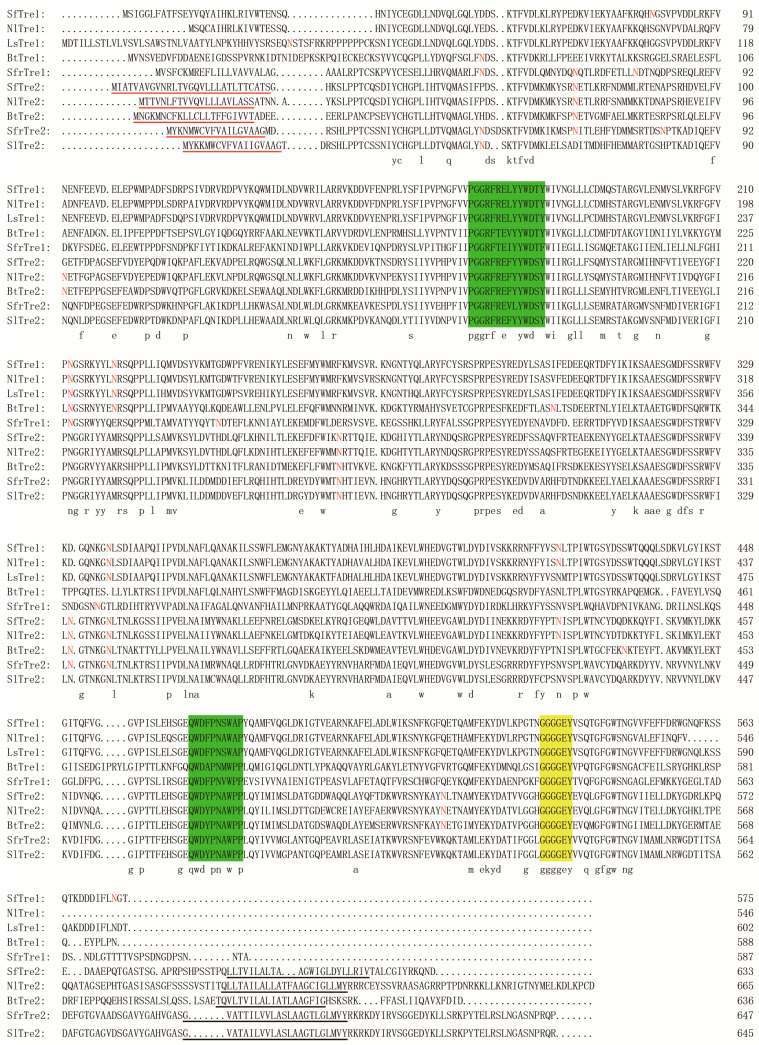
Multiple sequence alignment of insect trehalases was performed using DNAMAN 7. Identical amino acid residues are indicated by the letters below the sequence. The signal peptides are underlined in red, and the transmembrane regions are underlined. The trehalase signature motifs are shadowed in green, and the glycine-rich regions are shadowed in yellow. The *N*-glycosylation sites are in red. Abbreviations: NlTre1 and NlTre2 from *Nilaparvata lugens* (ACN85420 and ACV20872), LsTre1 from *Laodelphax striatellus* (AFL03409), BtTre1 and BtTre2 from *Bemisia tabaci* (AFV79626 and AFV79627), SfrTre1 and SfrTre2 from *Spodoptera frugiperda* (ABE27189 and ACF94698), SlTre2 from *Spodoptera litura* (ADA63845).

**Figure 2:**
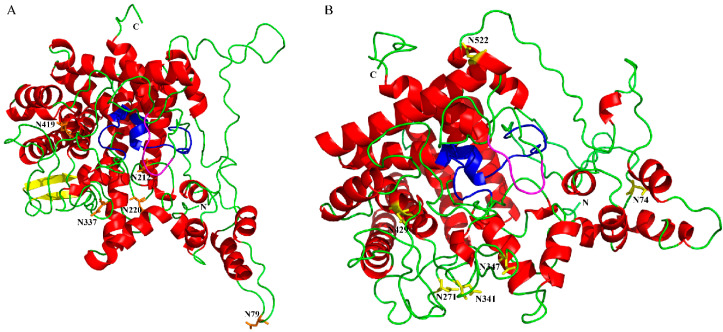
Three-dimensional model predictions generated by SWISS-MODEL homology modeling were performed using the crystal structure of periplasmic trehalase (PDB ID: 2wyn.1.A) as the template. (**A**) *Sf*Tre1 model; (**B**), *Sf*Tre2 model. α-helices are indicated in red. β-pleated sheets are indicated in yellow. Random coils are indicated in green. The signature motifs are indicated in blue. The glycine-rich region is indicated in purple.

We chose 6 species of different insects to verify the reliability of the alignment. Multiple sequence alignment of Tre proteins showed a significant sequence similarity (Figure 1). The sequence alignment also indicated that SfTre1 and SfTre2 proteins contain 2 typical Tre conserved signature motifs, respectively—PGGRFRELYYWDTY, QWDFPNSWAP, and PGGRFREFYYWDSY, QWDYPNAWPP. Both have a glycine-rich region—GGGGEY that is highly conserved among insect species (Figure 1). In addition, the SfTre2 protein possesses a signal peptide and transmembrane region, which is an important characteristic of Tre2. Furthermore, it was observed that SfTre1 has 6 potential *N*-glycosylation sites (amino acid residues 79, 212, 220, 337, 419, and 573), and SfTre2 also has six sites (amino acid residues 74, 271, 341 347, 429, and 522) (Figure 1). SWISS-MODEL homology modeling indicated that the three-dimensional (3D) structure of *Sf*Tre1 contains 22 α-helices, and 2 β-pleated sheets, whereas that of *Sf*Tre2 contains only 23 α-helices (Figure 2A,B).

The amino acid sequence encoded by both *SfTre*s in *S. furcifera* was aligned using the BLAST program. The results showed that *Sf*Tre1 shared high similarity with other insect Tre1 proteins, such as 95%, 92%, and 63% identity with *L. striatellus* (AFL03409), *N. lugens* (ACN85420), and *A. lucorum* (AGK89798), respectively. Similarly, *Sf*Tre2 shared 90%, 85%, 66%, and 63% identity with Tre2 of *L. striatellus* (AFL03410), *N. lugens* (ACV20872), *Bemisia tabaci* (AFV97627), and *A. lucorum* (AGL34007), respectively. In addition, the identity between *Sf*Tre1 and *Sf*Tre2 was 40%.

**Figure 3:**
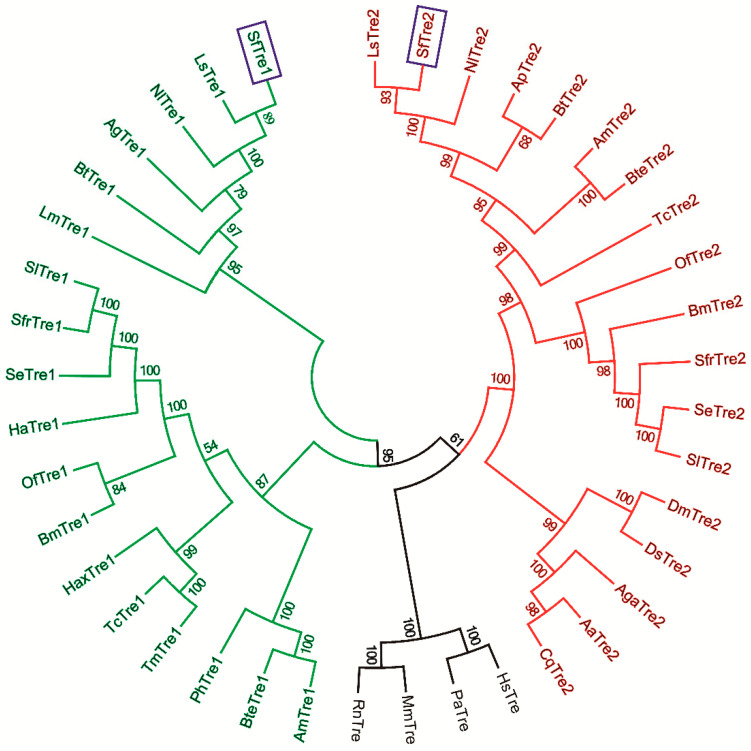
Phylogenetic tree of Tres in insects and other organisms. Organisms with the associated GenBank accession numbers are as follows: *Acyrthosiphon pisum* (Ap), *Aphis glycines* (Ag), *Bemisia tabaci* (Bt), *Laodelphax striatellus* (Ls), *Nilaparvata lugens* (Nl), *Bombyx mori* (Bm), *Helicoverpa armigera* (Ha), *Omphisa fuscidentalis* (Of), *Spodoptera exigua* (Se), *Spodoptera frugiperda* (Sfr), *Spodoptera litura* (Sl), *Apis mellifera* (Am), *Bombus terrestris* (Bte), *Pimpla hypochondriaca* (Ph), *Harmonia axyridis* (Hax), *Tenebrio molitor* (Tm), *Tribolium castaneum* (Tc), *Aedes aegypti* (Aa), *Anopheles gambiae* (Aga), *Culex quinquefasciatus* (Cq), *Drosophila melanogaster* (Dm), *Drosophila simulans* (Ds), *Locusta migratoria manilensis* (Lm), *Homo sapiens* (Hs), *Mus musculus* (Mm), *Pongo abelii* (Pa), and *Rattus norvegicus* (Rn). GenBank IDs are as follows: ApTre2 (XP_001949459), AgTre1 (AFJ00065), BtTre1 (AFV79626), BtTre2 (AFV79627), LsTre1 (AFL03409), LsTre2 (AFL03410), NlTre1 (ACN85420), NlTre2 (ACV20872), BmTre1 (BAA13042), BmTre2 (BAE45249), HaTre1 (AJK29979), OfTre1 (ABO20846), OfTre2 (ABO20845), SeTre1 (ABY86218), SeTre2 (ABU95354), SfrTre1 (ABE27189), SfrTre2 (ACF94698), SlTre1 (ADA63846), SlTre2 (ADA63845), AmTre1 (XP_393963), AmTre2 (NP_00110614), BteTre1 (XP_003400853), BteTre2 (XP_003393687), PhTre1 (CAD31109), HaxTre1 (ADH94051), TmTre1 (BAA01951), TcTre1 (XP_973919), TcTre1 (XP_972610), AaTre2 (EAT38444), AgaTre2 (XP_320471), CqTre2 (XP_001847934), DmTre2 (ABH06695), DsTre2 (ABH06710), LmTre1 (ACP28173), HsTre (NP_009111), MmTre (NP_067456), PaTre (XP_002822604), and RnTre (NP_001129613).

A phylogenetic tree was constructed based on the known amino acid sequences of Tres from other species by using the neighbor-joining method in MEGA 6.06 (Figure 3). The result revealed that Tre1 and Tre2 of all insects originated from the same starting point; however, they were clearly divided into two branches. As anticipated, *Sf*Tre1 and *Sf*Tre2 were extremely close to those from other planthoppers. Notably, they were grouped with other well-known Hemiptera insects, such as *L. striatellus*, *N. lugens*, *Acyrthosiphon pisum*, and *Aphis glycines*.

### 3.2. Spatiotemporal Expression of SfTre1 and SfTre2

**Figure 4:**
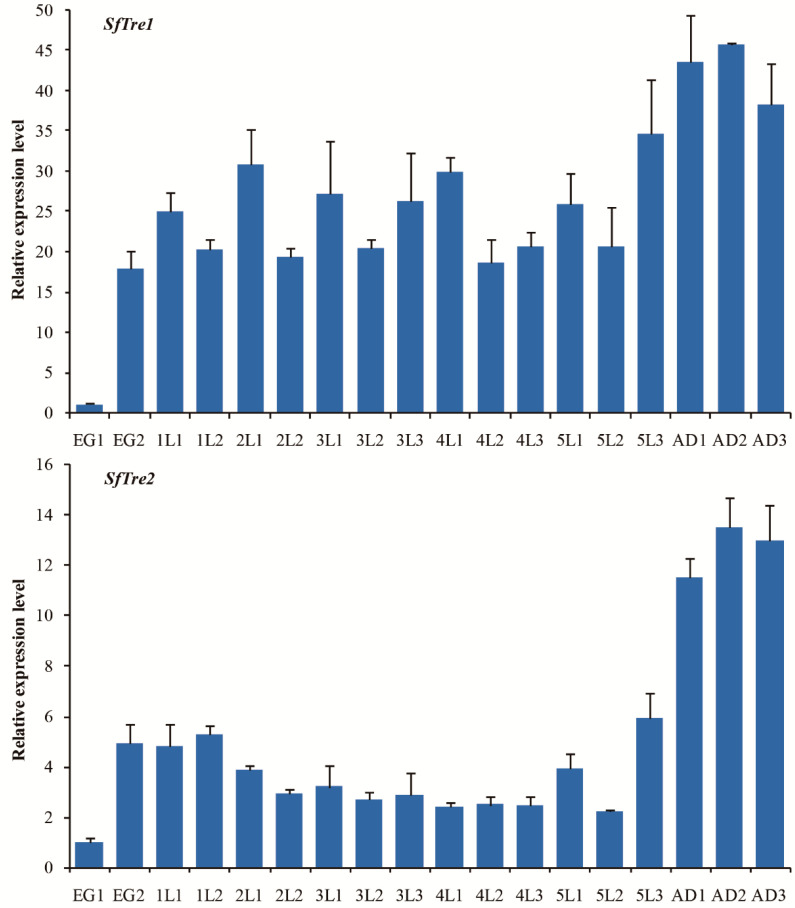
Expression profiles of *SfTre1* and *SfTre2* at different developmental stages of *S. furcifera*. *Sf18S* was used as an internal reference gene. Relative expression was determined based on the value of the lowest expression level, which was arbitrarily set to 1. Data are represented as means ± standard error of three biological replicates. The age in days of the insects is shown; for example, EG1 indicates the first day of eggs; lL1 indicates the first day of a first-instar nymph; AD1 indicates the first day of adults.

The expression profiles of both *SfTre*s at different developmental stages of *S. furcifera*, including egg, 1–5 instar nymph, and adult, were analyzed; the results are shown in Figure 4. *SfTre*s were continuously expressed at different developmental stages. In detail, the *SfTre1* expression level was relatively high after each molting, declined during the interval of the molting phase, and increased before the next molting. However, following eclosion, its expression level significantly increased and reached the maximum in day two adults. Compared with *SfTre1*, the expression level of *SfTre2* initially decreased and then increased. In particular, its expression level sharply increased in *S. furcifera* adults.

**Figure 5:**
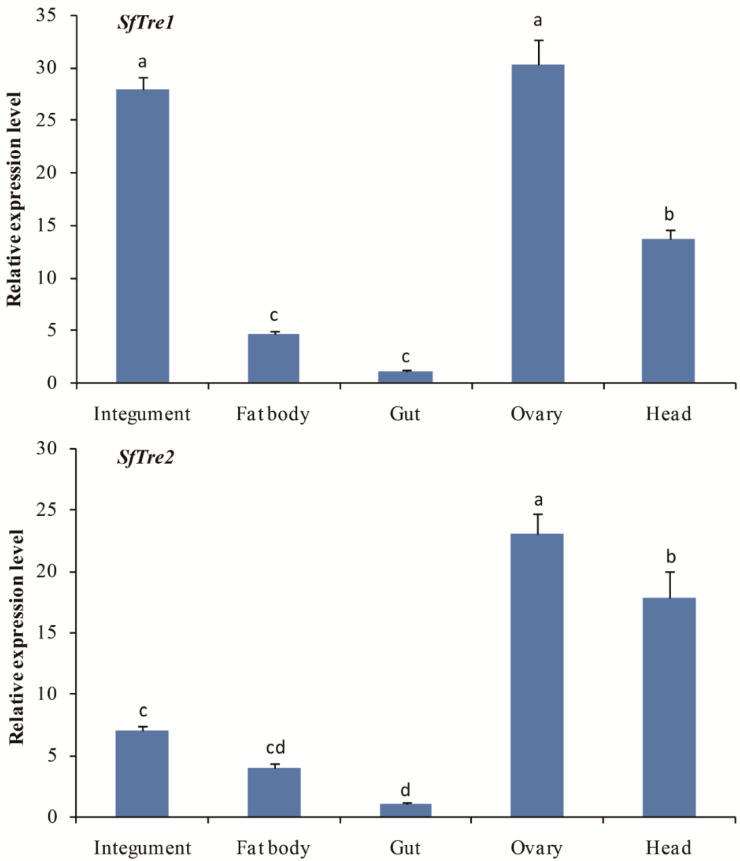
Expression profiles of *SfTre1* and *SfTre2* in different tissues of *S. furcifera*. *Sf18S* was used as an internal reference gene. The relative expression level was calculated based on the value of the lowest expression, which was arbitrarily set to 1. Data are presented as means ± standard error of three biological replicates. Different lowercase letters above the bars indicate significant differences (*p* < 0.05, Duncan’s multiple range test in one-way analysis of variance).

The mRNA expression levels of *SfTre1* and *SfTre2* in five different tissues of *S. furcifera* were evaluated by qRT-PCR. *SfTre1* was primarily expressed in the integument and ovary, followed by the head, and the lowest expression was observed in the fat body and gut (Figure 5). *SfTre2* showed the highest expression in the ovary, followed by the head, epidermis, and fat body, and it was the lowest in the gut (Figure 5).

### 3.3. Effects of RNAi

#### 3.3.1. Effects of RNAi on the expression levels of SfTre1 and SfTre2

**Figure 6:**
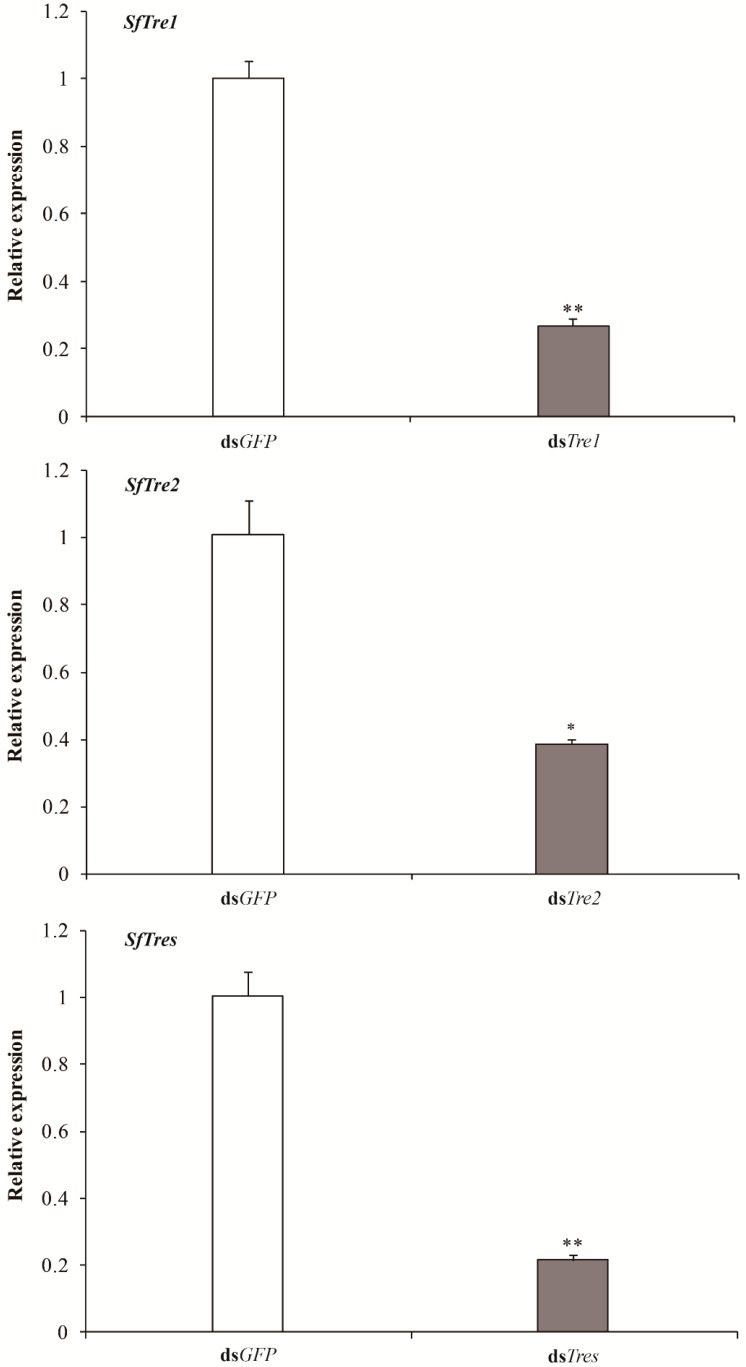
Relative transcript levels of *SfTre*s following RNAi. *Sf18S* was used as an internal reference gene. Data are presented as means ± standard error of three biological replicates. * indicates significant differences at the *p-*value of < 0.05 by independent sample *t*-test. ** indicates highly significant differences at the *p*-value of < 0.01. *SfTres*, *SfTre1* + *SfTre2*; ds*Tres*, ds*Tre1* + ds*Tre2*.

In RNAi, dsRNAs synthesized in vitro were injected into day one fifth-instar nymphs to verify the functional effect of *SfTre1 and SfTre2*. The expression levels of the target genes in *S. furcifera* at 72 h after injection were detected using qRT-PCR. The analysis indicated that all transcripts of the two *Tre* genes were significantly inhibited. In particular, the mRNA expression level of the ds*Tre1*, ds*Tre2*, and ds*Tres* treatment groups was significantly lower than that of the dsGFP control group, with a reduction of 73%, 62%, and 79%, respectively (Figure 6).

#### 3.3.2. Effects of RNAi on the Survival Rates

**Figure 7:**
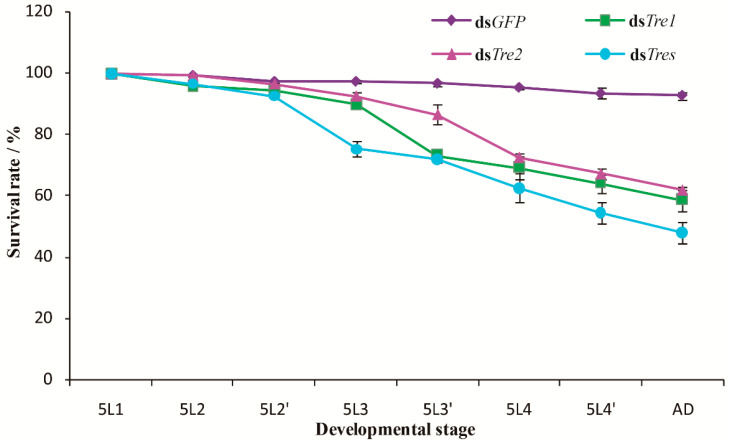
Survival rates of *S. furcifera* following RNAi. Data are presented as means ± standard error from three biological replicates with 50 insects in each group. The age in days of the insects is indicated; for example, 5L1 indicates the first day of fifth-instar nymphs; 5L2 and 5L2′ represent the two 12 h in the first day. AD, adults; ds*Tres*, ds*Tre1* + ds*Tre2*.

Following the successful injection of dsRNAs of the two *SfTre* genes, the survival of the insects subjected to RNAi was continuously recorded (Figure 7). Compared with the control group, the survival rate of the insects in the treatment group began to decrease significantly on day three after injection and continued to decrease with time. On day five, the survival rate of the insects injected with ds*Tre1* was 58%, whereas that of insects injected with ds*Tre2* was 62%. In addition, following the injection of a two gene dsRNA mixture, the survival rate of insects was 48%, which was lower than that following the injection of a single gene dsRNA. Therefore, the interference effect of mixed genes was superior to that of single gene.

#### 3.3.3. Effects of RNAi on Phenotypes

**Figure 8:**
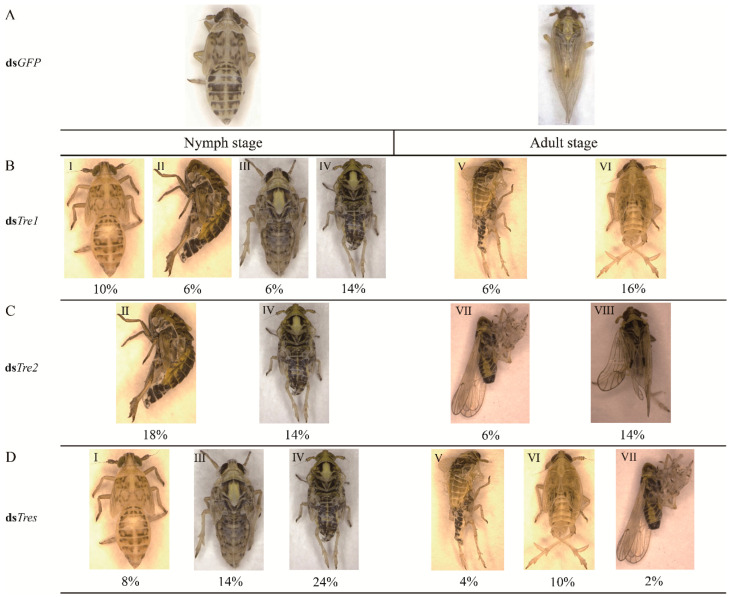
Representative phenotypes of *S. furcifera* caused by RNAi targeting different dsRNAs. (**A**), ds*GFP*-injected group; (**B**), ds*Tre1*-injected group; (**C**), ds*Tre2*-injected group. (**D**), ds*Tres*-injected group. All abnormal insects in the two stages (nymph and adult stages) were classified into eight types. The percentage below each type of abnormal insect phenotype indicates the corresponding proportion of abnormal insects in the injected group. ds*Tres*, ds*Tre1* + ds*Tre2*.

Following the successful injection of dsRNAs of the two *SfTre* genes, the insects in the nymph and adult stages exhibited various abnormal phenotypes (Figure 8). Four malformed phenotypes were observed in the nymph stage: the cuticle of the injected nymphs was not well hardened and looked transparent (I); the body size of the injected nymphs was significantly smaller than that of the normal ones (II); the old cuticle on the head and thorax slightly split open (III); the injected nymphs partially shed their old cuticle, but the insect body remained encased (IV). All II, III, and IV nymphs with the deformed phenotypes died (Figure 8). Furthermore, during the adult stage, four malformations were observed: the injected nymphs could exuviate the old cuticle to become adults, but the wings were abnormal (V); the body size of the injected adults decreased (VI); injected adults emerged successfully, but they could not extricate their old appendages (VII); the wings were malformed (VIII, Figure 8). These results indicated that the knockdown of both *SfTre*s seriously threatened insect normal growth and development, thereby causing the death of *S. furcifera*.

## 4. Discussion

*S. furcifera* is a serious pest affecting the rice industry in some Asia-Pacific countries. The application of chemical pesticides has extensively been the primary way to control *S. furcifera* [3,9]. However, the long-term use of insecticides will result in pest resistance, kill natural enemy insects, and pollute the environment [37,38]. Therefore, there is an urgent need to develop green control strategies for *S. furcifera* management.

Chitin is a major component of the cuticular exoskeleton and peritrophic membrane tissues, and it plays an essential role in insect development and metamorphosis. Insect chitin biosynthesis is a complex, dynamic process regulated by several enzymes [12,39]. Previous studies have suggested that the inhibition of several enzymes in insects could result in molting defects and high mortality [40,41,42,43]. Tre is a pivotal enzyme involved in the hydrolysis of trehalose in almost all tissues in distinct forms. In addition, Tre is commonly found in several organisms, including bacteria, algae, yeast, fungi, plants, nematodes, and insects, but not in mammals [44]. Therefore, Tre is a promising target for pest control. In the present study, we cloned and identified two *Tre* genes from *S. furcifera*—*SfTre1* and *SfTre2*. Phylogenetic analysis showed that the two *Tre* genes from *S. furcifera* shared the highest homology to those from *L. striatellus*. Furthermore, structural domain analyses demonstrated that these two genes shared some characteristic sequences with little difference, including two conserved signature motifs and a highly conserved glycine-rich region (GGGGEY); these characteristic sequences are unique to Tre and do not exist in other proteins [45,46]. However, we found that *Sf*Tre2 has a signal peptide of 29 amino acids at the N-terminus and a transmembrane region at the C-terminus, which is absent from *Sf*Tre1. Different results have been observed in *Laodelphax striatellus*, *Spodoptera litura*, and *Apolygus lucorum*, where the Tre1 also contains a signal peptide [30,47,48]. Previously, Tre1 has been purified from the egg homogenates, hemolymph, and goblet cell cavity in the midgut of some insects, whereas Tre2 has been found in the ovarian cells, follicular cells, spermatophore, flight muscles, brain, midgut, and thoracic ganglia [17,20,49,50,51]. Based on these results, we infer that the two *Tre* genes from *S. furcifera* could serve different functions.

Based on the expression levels of *SfTre1* and *SfTre2* genes at different developmental stages, the transcription level of *SfTre1* was found to repeat periodically in each molting cycle. In particular, *SfTre1* showed the highest expression level after molting, which decreased during the interval of each molting and increased before the next molting. The expression patterns of *SfTre1* are quite similar to those of *SfCHS1* and *SfUAP* in *S. furcifera* [34,35]. Furthermore, the expression levels of *SfTre1* and *SfTre2* were enriched in the egg and adult stages of *S. furcifera*, suggesting that their expression was associated with energy demand and chitin biosynthesis during highly metamorphic development. Previous studies have confirmed that the expression of insect *Tre* genes possesses tissue specific. *H. armigera Tres* are ubiquitous in detected tissues. In particular, *HaTre1* shows high expression levels in the midgut, integument, and head, whereas *HaTre2* shows high levels in the head [52]. In *A. lucorum*, the highest expression level of *Tre-1* is observed in the ovary and malpighian tubules, whereas that of *Tre-2* is observed in the flight muscle, fat body, and brain [48]. Moreover, *Tres* are widely distributed in various tissues of *G. pyloalis*, and *GpTre1* and *GpTre2* demonstrate the highest transcription levels in the head [32]. Meanwhile, in *Diaphorina citri*, the highest expression level of *Tre1-1* was noted in the head and integument, and that of *Tre1-2* was noted in the wing; moreover, the highest expression level of *DcTre2* was observed in the integument and fat body [53]. These results indicate that insect *Tres* play different roles in varying tissues. Our tissue transcription pattern analysis indicated that *SfTre1* and *SfTre2* showed the highest expression levels in the ovary. Insect ovaries are critical sites for egg cell formation. The high expression levels of *SfTre1* and *SfTre2* observed in the ovary may be attributed to high energy demand during egg cell development. Moreover, the abundant expression of *SfTre1* in the integument indicates that it may supply the nutritional sugar required in chitin biosynthesis. This result is consistent with previous studies on other insects, such as *Spodoptera exigua* and *Spodoptera litura* [44,47]. In the present study, *SfTre1* and *SfTre2* also exhibited high transcription levels in the head. The insect head is the anterior sensory and feeding center [54].

RNAi can induce targeted gene silencing, and it has successfully been used for evaluating functional genes in various insects [55,56,57,58,59,60]. Previous studies have shown that in *S. exigua*, the knockdown of *SeTre1* and *SeTre2* increases the mortality and abnormality rates and significantly decreases *CHS* expression levels and chitin content [44]. In *N. lugens*, ds*NlTres* injection represses the expression levels of genes related to the chitin metabolism pathway, including hexokinase (*HK*), glucose-6-phosphate isomerase (*G6PI*), chitinase, and *CHS*, thereby causing molting deformities and resulting in high mortality [61]. Moreover, the silencing of five *Tre* genes in *Tribolium castaneum* results in a decrease in the transcription levels of genes involved in the chitin biosynthesis pathway, including *HK*, *G6PI*, *CHS*, *TPS*, glycogen phosphorylase, glycogen synthase, phosphofructokinase, and fructose-6-phosphate transaminase and an increase in the lethality and malformation rates [62]. These results indicate that *Tre* plays a vital role during insect metamorphic development and survival. In our study, RNAi was used to investigate the biological function of *SfTre1* and *SfTre2* during the growth and development of *S. furcifera*. When fifth-instar nymphs on day one were injected with ds*Tre1* and ds*Tre2*, qPCR results showed the expression levels of *SfTre1* and *SfTre2* were significantly hindered. The RNAi-mediated downregulation of *SfTre1* and *SfTre2* transcription ultimately resulted in various malformed phenotypes and high mortality. Our results were in accordance with previous data obtained in *G. pyloalis* and *A. pisum* [32,63]. We infer that the inhibition of *Tres* may prevent the hydrolysis of trehalose from generating glucose, thereby affecting chitin formation. In summary, our preliminary results may lay a foundation for further exploring the regulatory mechanism of *S. furcifera* Tre and provide a potential target for planthopper control.

## 5. Conclusions

In the present study, we successfully identified and characterized two *SfTre* genes from *S. furcifera*. Silencing of *SfTre1* and *SfTre2* via RNAi severely impeded the normal development of the insect. In particular, the knockdown of *SfTre*s (*SfTre1* and *SfTre2*) gene expression by RNAi resulted in a mortality rate of >50%, which indicated the crucial roles played by these genes in the development of *S. furcifera*. Our findings could provide potential target genes for RNAi-based *S. furcifera* control.

## Figures and Tables

**Table 1:** Primers used in the study.

Gene	Application Type	Primer Name	Primer Sequence (5′-3′)
*SfTre1*	5′ RACE	Tre1-R51	CTGCCATTGTGCTGTCTCTTG
		Tre1-R52	AGTCACCTTCGCAATAGATG
	Validation PCR	SfTre1-v-F	TCAAAATGTCGATTGGCGG
		SfTre1-v-R	CTCACACTTATCACGTACC
	qRT-PCR	SfTre1-q-F	GACTTCTGCTATGTGATATGC
		SfTre1-q-R	GCTGTCCACCATCTGAATA
	dsRNA synthesis	dsSfTre1-F	*TAATACGACTCACTATAGGG*CGGACTTCTGCTATGTGATA
		dsSfTre1-R	*TAATACGACTCACTATAGGG*AACGAACCATCTTGAACTGA
*SfTre2*	PCR1	SfTre2-F1	ATGATGCTGAGGACTGAGA
		SfTre2-R1	ACTCTTCAACAATGGTCACA
	PCR2	SfTre2-F2	AACTACTACGGAGAACTGAAG
		SfTre2-R2	TGTGAGATTGTATGCCTTGT
	5′ RACE	Tre2-R51	TTCCACAGTAGATTGAGTTG
		Tre2-R52	CATTCTCAGTCCTCAGCATC
	Validation PCR	SfTre2-v-F	TCCATCAAGCATGATAGC
		SfTre2-v-R	TACCCAGTTGTTCTAATCAT
	qRT-PCR	SfTre2-q-F	GTGGTTGGATGCTGTTACTA
		SfTre2-q-R	GAGATGTTTGTCGGGTAGAA
	dsRNA synthesis	dsSfTre2-F	*TAATACGACTCACTATAGGG*TACTGTTGCTGTTGGTGTTA
		dsSfTre2-R	*TAATACGACTCACTATAGGG*CGTCTTCACATCATCCTTCA
18S rRNA	qRT-PCR	Sf18S-q-F	CGGAAGGATTGACAGATTGAT
		Sf18S-q-R	CACGATTGCTGATACCACATAC
GFP	dsRNA synthesis	dsGFP-F	*TAATACGACTCACTATAGGG*AAGGGCGAGGAGCTGTTCACCG
		dsGFP-R	*TAATACGACTCACTATAGGG*CAGCAGGACCATGTGATCGCGC

Abbreviations: RACE: Rapid Amplification of cDNA Ends; PCR: Polymerase chain reaction; qRT-PCR: Quantitative real-time PCR; dsRNA: Double-stranded RNA; GFP: Green fluorescent protein.

**Table 2:** Sequence information of *SfTre1* and *SfTre2*.

Gene	Length of cDNA (bp)	Number of Amino Acids	Molecular Weight (kDa)	Isoelectric Point	Accession Number
*SfTre1*	3700	575	67.01	5.44	MG869613
*SfTre2*	2757	633	72.82	5.65	MG869614

Abbreviations: cDNA: complementary DNA.

## Data Availability

The data were deposited in GenBank with accession numbers MG869613 and MG869614.
